# Detection of a New Large Free Core Nutation Phase Jump

**DOI:** 10.3390/s22165960

**Published:** 2022-08-09

**Authors:** Zinovy Malkin, Santiago Belda, Sadegh Modiri

**Affiliations:** 1 Pulkovo Observatory, St. Petersburg 196140, Russia; 2 University of Alicante VLBI Analysis Centre (UAVAC), University of Alicante, 03080 Alicante, Spain; 3 Image Processing Laboratory (IPL)—Laboratory of Earth Observation (LEO), University of Valencia, 46980 Valencia, Spain; 4 Department Geodesy, Federal Agency for Cartography and Geodesy (BKG), 60322 Frankfurt am Main, Germany

**Keywords:** Earth’s rotation, celestial pole motion, free core nutation (FCN), FCN phase variations, VLBI

## Abstract

We announce the detection of a new large jump in the phase of the free core nutation (FCN). This is only the second such large FCN phase jump in more than thirty years of FCN monitoring by means of a very long baseline interferometry (VLBI) technique. The new event was revealed and confirmed by analyzing two FCN models derived from a long-time series of VLBI observations. The jump started in 2021 and is expected to last until the late fall of 2022. The amplitude of the phase jump is expected to be approximately 3 rad, which is as much as 1.5 times larger than the first phase jump in 1999–2000. A connection of the new FCN phase jump with the recent geomagnetic jerk started in 2020 is suggested.

## 1. Introduction

The movement of Earth’s rotation axis in space consists of several forced and free components, such as precession and nutations. One of these components is the free core nutation (FCN), which is one of free Earth’s rotational modes with retrograde frequency caused by the misalignment of the rotational axes of the Earth’s mantle and outer liquid core [1]. Although the FCN was theoretically predicted more than a century ago as a common rotational mode of a rotating body with an ellipsoidal solid outer shell and inner liquid core, its experimental detection only became possible with the beginning of the implementation of a very long baseline interferometry (VLBI) method for monitoring the full set of Earth orientation parameters (EOP). The EOP includes parameters of the polar motion, universal time (or Earth’s rotation speed), and celestial pole offsets (CPOs), which are the differences between observed coordinates of the Earth’s celestial pole in the celestial reference system and the standard precession–nutation model IAU 2006/2000A [2,3,4].

The FCN is the largest and poorly predictable part of CPO, and its investigation is an important scientific and applied task. First, the FCN parameters determined from observations provide valuable, sometimes unique, information about processes in the Earth’s interior. From a practical point of view, the accurate modeling of the FCN term, including prediction, is necessary to compute the CPO with an accuracy compatible with modern VLBI observations for near-real-time applications and EOP prediction [5].

Numerous analyses of the VLBI-derived CPO series showed that both FCN amplitude and phase are highly variable. Causes of this variability need to be identified and predicted with high accuracy, which is an indispensable requirement to fulfill the stringent targets of the Global Geodetic Observing System (GGOS) of the International Association of Geodesy (IAG) [6]. In this study, we used the latest combined CPO series obtained by the International Earth Rotation and Reference Systems Service (IERS) [7] and International VLBI Service for geodesy and astrometry (IVS) [8] to perform investigation. This allowed us to reveal the newest features in the FCN amplitude and phase variations. Section 2 describes the method of computation and the obtained results, which are further discussed in Section 3.

## 2. FCN Series: Computation and Analysis

This study is based on the analysis of long-term CPO series as observed by VLBI. These series were used to evaluate two empirical FCN models. The former FCN model, called “ZM3”, was constructed as follows. First, the ZM2 CPO model was constructed by the Gaussian smoothing of the IVS combined EOP series (https://ivscc.gsfc.nasa.gov/products-data/product-tables/bkg-products-eops.html (accessed on 10 March 2022)) [9]. Two-year forward prediction was also computed for this series using the autoregression method of the order of 10. After that, the ZM3 FCN model (series) was evaluated using the following expressions:

(1) $$\begin{array}{ccc} dX & = & A_{c}\cos\varphi -A_{s}\sin\varphi +X_{0}\;,\\ dY & = & A_{c}\sin\varphi +A_{s}\cos\varphi +Y_{0}\;, \end{array}$$

The model parameters $A_{c}$, $A_{s}$, $X_{0}$, and $Y_{0}$ were adjusted by the least square method for running 431-day intervals with a 1-day shift. The length of the interval was chosen to be the nearest odd number of days to the nominal FCN period $P_{FCN}$= −430.21 solar days as recommended by the IERS Conventions (2010) [4]. Other parameters in Equation (1) are: $\varphi =2\pi /P_{FCN}\left(t-t_{0}\right)$, $t_{0}$ = J2000.0, and *t* is the epoch at which observed dX and dY values are given. Each pair of equations in Equation (1) corresponds to one CPO epoch given in the combined IVS solution. The model parameters $A_{c}$, $A_{s}$, $X_{0}$, and $Y_{0}$ were computed during the middle epoch of each 431-day interval. Thus, the resulting FCN parameters are also given with one-day steps.

The FCN contribution to the celestial pole motion at the given epoch is computed by using Equation (1) without the shift terms $X_{0}$ and $Y_{0}$.

(2) $$\begin{array}{ccc} dX_{FCN} & = & A_{c}\cos\varphi -A_{s}\sin\varphi \;,\\ dY_{FCN} & = & A_{c}\sin\varphi +A_{s}\cos\varphi \;, \end{array}$$

Then, the amplitude *A* and phase *P* of the FCN signal can be computed using the following expressions:

(3) $$\begin{array}{ccc} A_{FCN} & = & \sqrt{dX_{FCN}^{2}+dY_{FCN}^{2}}\;=\;\sqrt{A_{c}^{2}+A_{s}^{2}}\;,\\ P_{FCN} & = & \arctan\frac{A_{c}}{A_{s}}\;, \end{array}$$

Since the whole ZM2 series, including two-year prediction up to epoch 2024.4, was used to compute the ZM3 series, the latter also includes prediction. The length of the ZM3 prediction is approximately 1.5 years, which is determined as the length of the ZM2 prediction minus a half of the length of the running interval used for the computation of the ZM3 model. Therefore, the ZM3 series ends at epoch 2023.8.

**Figure 1:**
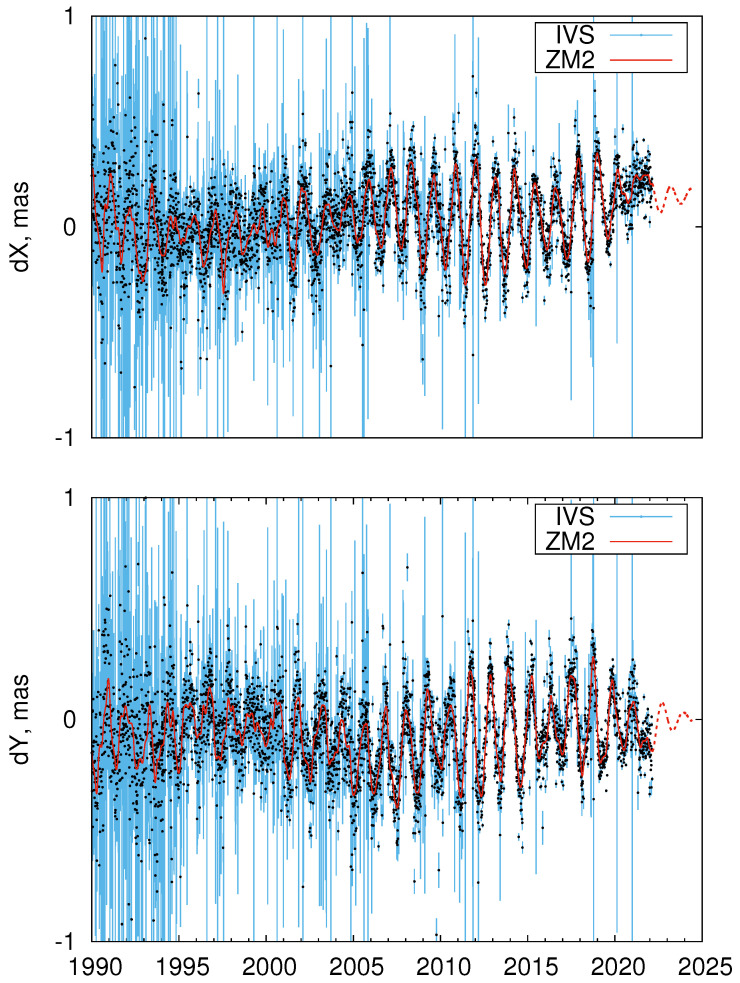
ZM2 (red line) and IVS (black points with blue error bars) CPO series. Dashed red line shows ZM2 prediction.

Figure 1 shows the ZM2 CPO model along with the underlying IVS CPO series. Two epochs of abrupt improvement in the accuracy of the VLBI-based EOP series can be noticed in May 1993, when the NEOS-A observing program started, and in the beginning of 2002, when the IVS observing programs R1 and R4 started [10].

The second FCN model used in this study, called “SB”, was constructed in a similar way to the ZM3 model with a few exceptions [11,12]:

(a) SB FCN series is based on the IERS C04 EOP series (https://datacenter.iers.org/eop.php (accessed on 15 March 2022)) [13];
(b) FCN period is assumed to be equal to −430.0027 solar days as obtained by [14] from VLBI observations;
(c) The length of the sliding window is set to 400 days.

**Figure 2:**
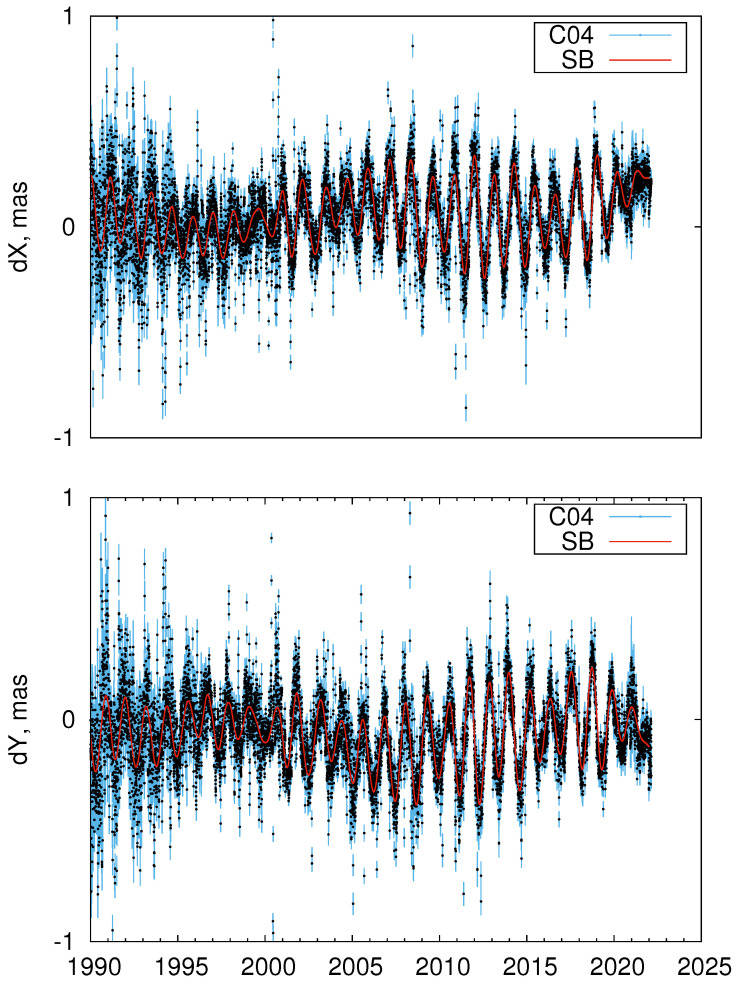
SB (red line) and IERS C04 (black points with blue error bars) CPO series.

Figure 2 shows the SB CPO model along with the underlying IERS C04 series.

**Figure 3:**
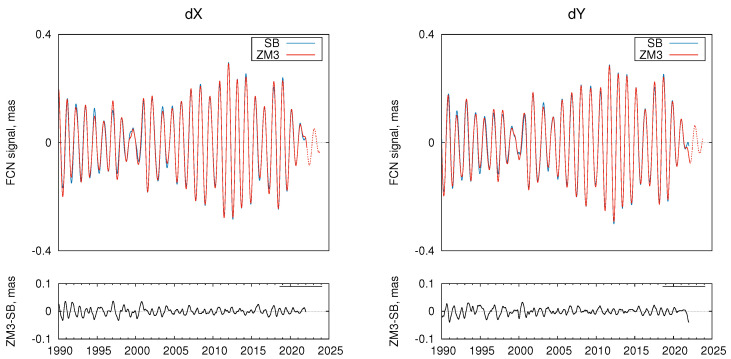
Upper panels: SB (blue line) and ZM3 (red line) FCN series; dashed red line shows ZM3 model prediction. Bottom panels: ZM3 minus SB model differences.

**Figure 4:**
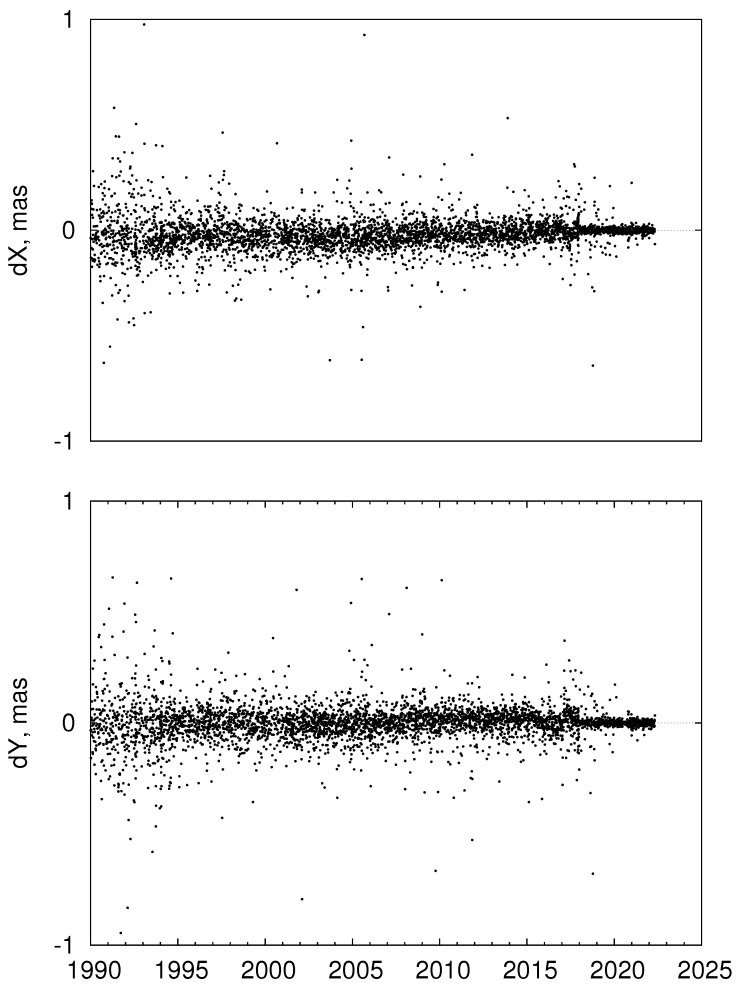
Differences between the IVS and IERS C04 CPO series (IVS minus IERS).

The two FCN models, ZM3 and SB, are compared in Figure 3. One can see that two models are very close except for the period before 2002 (start of the R1 and R4 IVS observing programs [10]) and the very end of the series. One of the main reasons between the discrepancies of the SB and ZM3 FCN models are the differences between the IERS C04 and IVS CPO series shown in Figure 4. For this comparison, C04 CPO values were linearly interpolated at the IVS CPO epochs. The differences between the IVS and IERS CPO series were discussed in more detail in [15].

The choice of the optimal window length deserves a separate discussion. There are several approaches proposed in the literature to solve this problem. During the construction of the ZM FCN models, it was considered important to have a window length multiple of the FCN period to eliminate a signal with this period from the amplitude and phase variations. The choice of window length of one FCN period was supposed a good trade-off between the degree of smoothing and temporal resolution. Belda et al. [11] derived the optimal window length of 400 days which provided the minimum of the combined error which includes both the error of the fit of the CPO series with FCN model and the uncertainty of the FCN model coefficients. Krásná et al. [14] used a 4-year sliding window without providing an explanation of the rationale.

The FCN series provided by the IERS EOP Product Center (https://ivsopar.obspm.fr/fcn/index.html (accessed on 28 March 2022)) is computed using a 7-year window length. Such a relatively large sliding window was selected to separate the FCN series from the retrograde annual signal [16]. The author computed the demodulation (beating) period for 6.7 years and suggested that an oscillation with such a period can be introduced in the FCN amplitude variations when using a much shorter window length. The window length of 6.7 years was recommended by [17] to compute their FCN model, which is a bit shorter than the window length used for the IERS FCN series.

Zhu et al. [18] also used wide an 8-year window for the computation of their FCN model to eliminate the retrograde annual signal. The authors also compared the amplitude variations for six-test FCN models with a window length of 3–8 years with a 1-year step. As expected, all the models showed a very similar FCN amplitude behavior with the same epochs of the maxima and minima of the FCN amplitude and the same peak-to-peak variations. The only effect that can be observed is the increasing degree of smoothness of the FCN amplitude series with the increasing window length, which is naturally expected.

A similar test was performed by [19]. The authors compared several FCN models computed with the length of the sliding window of 2, 3, 4, 5, and 6.7 years, and also found that the resulting series are in good agreement and mostly differ in the degree of smoothness.

Therefore, as the main goal of this work is the investigation of the large-scale FCN variations, there is no need to increase the length of the sliding window to mitigate the weak remaining signal. Increasing the window length to approximately 7 years is important for the analysis of the nutation terms close to the FCN frequency, but not for our task. More discussion on this point will be given below.

**Figure 5:**
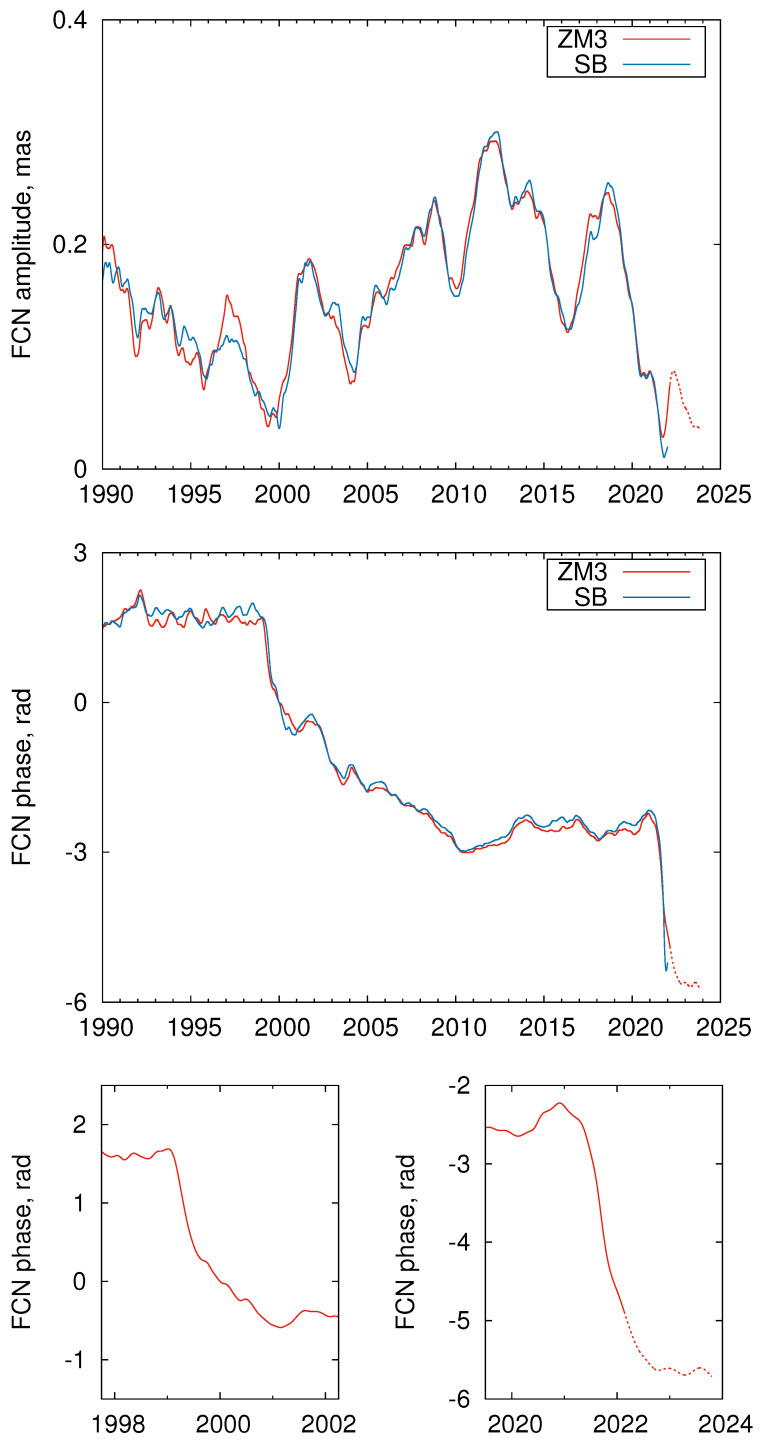
Variations of the FCN amplitude (**upper panel**) and phase (**middle panel**). The bottom zoomed panels show phase variations around two large phase jumps in the same scale.

Finally, the main results of our study are presented in Figure 5, which shows the FCN amplitude and phase variations derived from both ZM3 and SB FCN series. The phase variations are shown after subtracting the linear drift corresponding to the nominal FCN frequency. As can be expected from comparing the SB and ZM3 models shown in Figure 3, the amplitude and phase variations derived from two FCN models are close enough with larger differences observed before 2002 and at the end of the series.

The FCN phase variations, which are of primary interest for this work, coincide well. The phase variations before ∼2020 mostly correspond to the aforementioned results of other studies. However, the addition of the most recent CPO data allowed us to reveal a new phase jump similar to the previous large jump in 1999–2000, but of even larger amplitude, as can be seen in the zoomed bottom panels of Figure 5. It is also noticeable that the FCN phase is changing more sharply than observed during the period 1999–2000.

**Figure 6:**
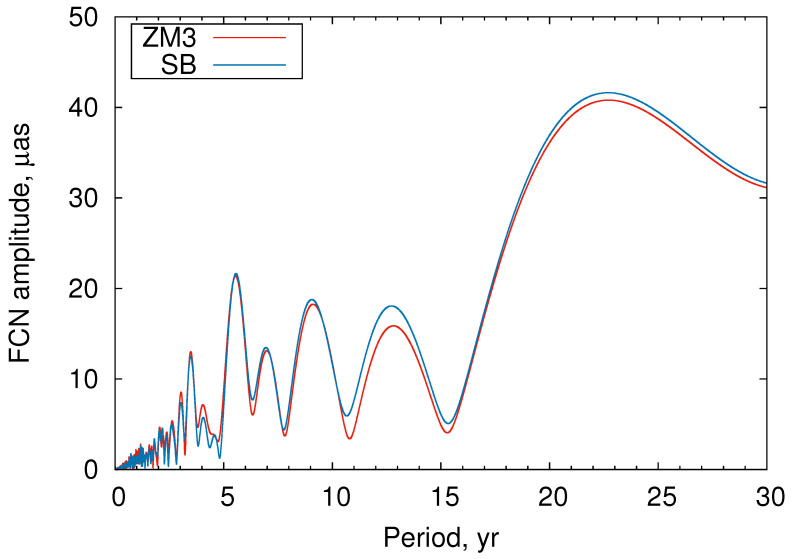
Spectrum of the FCN amplitude time series.

To check the conclusions made by [16,17,18] about the possible distortion of the FCN amplitude series caused by close retrograde annual oscillation, we computed the spectra (periodogram) of the FCN amplitude series (Figure 6). These data show no manifestation of the signal with a period of 6.7 years. There are several signals in the close frequency band, but no single one prevails. As for the signal with a period of 1 year, which might be caused by the remaining part of the retrograde annual oscillation, it is practically at the noise level. Therefore, we can conclude that using a window length of 400 or 430 days does not lead to introducing a detectable spurious signal in the FCN parameters’ variations.

## 3. Discussion and Conclusions

In this study, we detected a new large phase jump that occurred during the period 2021–2022. To improve the reliability of this result, we analyzed two different FCN series based on different combined CPO series. It is the second large FCN phase jump since the beginning of highly accurate CPO monitoring with the VLBI technique. The first large FCN phase jump observed in 1999–2000 had an amplitude of approximately two radians. According to the latest CPO predictions, the new FCN phase jump discussed in this study will last until late fall of 2022 and will have an amplitude of more than three radians. It is remarkable that, like the first large FCN phase jump observed in 1999–2000, the new jump occurred near the epoch of minimum FCN amplitude. This is very similar to the large Chandler wobble (CW) phase jump also observed at the epochs near the minima of the CW amplitude [20].

Unfortunately, this discovery cannot be extracted by using other recent FCN models, such as those computed by [18] and at the IERS EOP Product Center. The main reason is that these models do not last until the epoch of the FCN phase jump discussed in this paper. On the other hand, previous comparisons [11,14,15,18,19] showed that all known FCN series developed by various authors mostly differed in the degree of smoothing, which mainly depends on the length of the sliding window. It is naturally expected because all the FCN series are based on the same CPO series derived from the same VLBI observations, and the selection effect has a small impact on the final result. All the series show similar behavior in the sense of the FCN amplitude and phase large-scale variations, which are of primary interest to this study. For example, all the series show a similar minimum for the FCN amplitude and large FCN phase jump around 1999–2000. The advantage of using the model with a relatively short sliding window is its ability to detect abrupt FCN changes in a more timely manner.

Among the most probable sources of the FCN phase jumps, one can assume geophysical processes in the Earth’s core and at the core–mantle boundary. These processes can also cause variations in the geomagnetic field (GMF), particularly geomagnetic jerks (GMJ). Several studies have been devoted to investigating the interconnection between the FCN and GMF variations. The first evidence of a possible connection between the large FCN phase jump in 1999 and GMJ was presented by [21]. After that, only relatively small phase and/or phase rate jumps were observed [22,23]. More detailed investigations of this effect were conducted by [18,19,24,25,26]. Recently, [27] reported about newly detected signs of a start of a GMJ in 2020, which is likely connected with the FCN phase jump 2021–2022 detected in this work. Based on previous studies, this assumption looks highly reasonable.

Although many papers have been devoted to the analysis of the observed temporary correlation between the Earth’s rotation and GMF variations, no convincing theory directly connecting these phenomena has been developed. Moreover, most probably, there is no direct connection between them, and both FCN and GMF variations are caused by some common processes in the Earth’s interior.

Investigations of the FCN phase jumps and other variations are interesting for geophysics and are also practically important for CPO prediction. The largest effect can be expected near the epoch of a minimum of the FCN amplitude when the FCN oscillation is most probably less stable.

## Data Availability

IERS C04 CPO series is publicly available at https://datacenter.iers.org/eop.php (accessed on 15 March 2022). IVS CPO series is publicly available at https://ivscc.gsfc.nasa.gov/products-data/product-tables/bkg-products-eops.html (accessed on 10 March 2022). ZM FCN and CPO series are publicly available at http://www.gaoran.ru/english/as/persac/ (accessed on 10 March 2022). SB FCN series can be available from S.B. upon reasonable request.
